# Evaluating the care provision of a community-based serious-illness care program via chart measures

**DOI:** 10.1186/s12877-020-01736-z

**Published:** 2020-09-15

**Authors:** Christine E. Kistler, Matthew J. Van Dongen, Natalie C. Ernecoff, Timothy P. Daaleman, Laura C. Hanson

**Affiliations:** 1 Department of Family Medicine, University of North Carolina, 590 Manning Drive, CB #7595, Chapel Hill, NC 27599 USA; 2 Department of Internal Medicine, University of North Carolina, Chapel Hill, NC USA; 3 Cecil G. Sheps Center for Health Services Research, University of North Carolina, Chapel Hill, NC USA; 4 Division of General Internal Medicine, University of Pittsburgh School of Medicine, Pittsburgh, PA USA

**Keywords:** home-based primary care, home-based palliative care, community-based care, quality-of-care, serious-illness care

## Abstract

**Background:**

Although quality-of-care domains for home-based primary and palliative programs have been proposed, they have had limited testing in practice. Our aim was to evaluate the care provision in a community-based serious-illness care program, a combined home-based primary and palliative care model.

**Methods:**

Retrospective chart review of patients in an academic community-based serious-illness care program in central North Carolina from August 2014 to March 2016 (*n* = 159). Chart review included demographics, health status, and operationalized measures of seven quality-of-care domains: medical assessment, care coordination, safety, quality of life, provider competency, goal attainment, and access.

**Results:**

Patients were mostly women (56%) with an average age of 70 years. Patients were multi-morbid (53% ≥3 comorbidities), functionally impaired (45% had impairment in ≥2 activities of daily living) and 32% had dementia. During the study period, 31% of patients died. Chart review found high rates assessment of functional status (97%), falls (98%), and medication safety (96%). Rates of pain assessment (70%), advance directive discussions (65%), influenza vaccination (59%), and depression assessment (54% of those with a diagnosis of depression) were lower. Cognitive barriers, spiritual needs, and behavioral issues were assessed infrequently (35, 22, 21%, respectively).

**Conclusion:**

This study is one of the first to operationalize and examine quality-of-care measures for a community-based serious-illness care program, an emerging model for vulnerable adults. Our operationalization should not constitute validation of these measures and revealed areas for improvement; however, the community-based serious-illness care program performed well in several key quality-of-care domains. Future work is needed to validate these measures.

## Background

Of the 45 million adults aged 65 and older currently living in the US, approximately 2–4 million are considered homebound due to serious illness or disability and are unable to readily access office-based healthcare [1–3]. Models of home-based medical care, including both home-based primary care and palliative care are emerging to help to meet the healthcare needs of home-bound patients with serious medical illness [4–7]. One emerging model is combined primary and palliative care delivered in patients’ communities for patients with serious illness, called community-based serious-illness care. As community-based serious-illness care programs have grown, quality-of-care domains and standards have been proposed [8, 9]. Community-based serious-illness care programs will need to effectively determine the quality-of-care they provide [3, 10]. Operational gaps currently impede the ready adoption of quality-of-care measurement in community-based serious-illness care programs. The use of electronic health records have demonstrated several challenges to operationalizing, collecting, and reporting these measures in palliative care environments [11]. If community-based serious-illness care programs are to demonstrate value and ensure accountability in the care of vulnerable patients, further assessment needs to be done [12, 13]. Chart review can provide a way to assess quality of care in other fields [14, 15]. To better understand the current quality-of-care, we created operational definitions for selected quality-of-care measures and used them to evaluate a community-based serious-illness care program. The program has been evaluated for its impact in older adults with dementia, its impact on healthcare utilization, and in palliative care, but not in proposed quality-of-care [16, 17]. The aim of the study was to determine if we could operationalize proposed quality-of-care domains and then evaluate our program [8, 9].

## Methods

### Study design

As part of a program evaluation, we conducted a retrospective chart review to evaluate a community-based serious-illness care program, the REACH program (Reaching out to Enhance the care of Adults in their Communities and their Homes) based at an academic medical center. Data collection included chart reviews of all patients enrolled from August 2014 to March 2016. Two research assistants independently completed a subset of chart reviews of visit notes and patient data in the electronic health record (EHR), reconciled differences until reviews were consistent, and conferred regularly to prevent drift and maintain data quality. Using the EHR EPIC, the initial clinical visit note was templated into a semi-structured format, though other elements could be added separately to the note, and the plan of care was untemplated. Clinicians were not aware they were being evaluated during the time period of the study. A formal chart review guide was developed to standardize the data abstraction process.

### Community-based serious-illness care model

The REACH program was a community-based serious-illness care program that extended both medical and palliative care to seriously ill and medically complex adults in their homes or assisted living communities [16–18]. The REACH program was designed to meet the needs of patients and primary care providers and thus offered an array of services from short-term consultation to ongoing co-management to assuming primary care if needed. The REACH program was interdisciplinary, with a team comprised of [1]: clinicians (MD, DO, nurse practitioner) with expertise in geriatrics and/or palliative care [2]; a clinical pharmacist (PharmD) [3]; a care manager (Master’s of Social Work); and [4] a nurse coordinator (RN) who trained in the Guided Care model, a model designed to coordinate care across healthcare settings [19]. The program’s goals were for the patients to be seen at least quarterly, or more often as needed based on the evidence that frequent visits were needed to improve care outcomes [20]. Patients are seen by clinicians in their homes for an initial visit with support from the other team members remotely. The nurse coordinator occasionally accompanied the clinician to these visits and family members or caregivers could be present. The pharmacist and social worker would help provide input in medication management or social support resources, respectively, as needs identified by the visiting clinicians arose. The nurse coordinator would coordinate care with home health agencies, hospice, medication refills, radiologic orders and results, and orders for durable medical equipment. Patients had direct access to the nurse coordinator during business hours for acute issues and to an after-hour nurse triage system if needed. The nurse coordinator made infrequent visits to patient homes. Urgent visits could be made upon request.

### Participants & eligibility

Patients were eligible if they were seen for an initial visit by a REACH clinician in their home or assisted living community during the study period. Patients were referred by their primary care clinicians to enroll in REACH. Patients were eligible if they had become unable to access office-based care or had serious medical illnesses that made coordinated primary care challenging, were adults living within 30 miles of the medical campus, and agreed to home visits. Nursing home patients were not eligible.

### Study measures

Chart review collected demographic information and days enrolled in the program. We also quantified the degree of patients’ serious illness using the Charlson Comorbidity Index (CCI) [21]. The CCI is comprised of 19 specific medical conditions and their severity [21]. The CCI yields a score from 0 to 35, where higher numbers represent worse overall health, and was assessed from available records within three months after the REACH initial visit. We also assessed functional status via the presence of the Katz Instrumental Activities of Daily Living (IADLs) [22], and basic ADLs [23]. Higher IADLs and ADLs represent better function [22, 23]. IADLs measure more complicated aspects of daily function such as managing finances, medications, and transportation. ADLs measure basic functions such as toileting, walking, or eating. We recorded independence in IADLs and ADLs (Yes/No). Other measures such as hospitalization and palliative care measures are reported elsewhere [16, 18].

To assess the quality-of-care delivered by the community-based serious-illness care program, we operationalized standards based on seven of ten quality domains proposed by the National Home-Based Primary and Palliative Care Network [8]. We assumed that if a patient warranted admission into the community-based serious-illness care program that they should be assessed for all of these standards. The program was developed to adhere to standards of care. At the time of the program evaluation, no standards had been explicitly published, though the standards we used were very similar to current standards [24]. We attempted to find a standard that could be completed by chart review for each quality-of-care domain. Our team collaboratively mapped each of these standards onto the clinical visit notes. We were not able to operationalize three domains to chart review (patient education, patient and caregiver experience, and cost) and did not look at frequency of visits or other important quality metrics such as emergency department visits or hospitalizations, which need alternative methods of assessment. Though the proposed standards include more than those we assessed, we developed the following chart-based standards for seven domains, abstracted from the initial visit:

- *Medical assessment*: percent of initial visits with a) structured functional status assessment [22, 23], b) structured cognitive screening (via the Veterans Affairs St. Louis University Mental Status Exam (VA-SLUMS, range 0–30, with higher scores being better cognition) [25], c) assessment of communication barriers (e.g., confusion/sedation, dementia, language barrier, or other), and d) assessment of spiritual needs
- *Care coordination*: percent of initial visits with documented communication with EITHER a primary care clinician, specialty clinician, or REACH team social worker or pharmacist.
- *Safety*: percent of initial visits with a) medication review, b) assessment for falls, and c) assessment of behavioral issues
- *Quality of life*: percent of initial visits with comprehensive symptom assessment inclusive of pain, dyspnea, constipation, poor appetite, nausea, depressive symptoms, anxiety, fatigue, and psychosocial distress
- *Clinician competency*: percent of initial visits with a) an assessment or administration of the influenza vaccine, b) prescription for antidepressant among those with depression, c) opiate prescription (and bowel regimens), d) antipsychotic and benzodiazepine medication prescription (for each, lower frequency is better quality)
- *Goal attainment*: percent of initial visits with documented discussions of advance directives, goals of care, and surrogate decision-makers
- *Access*: percent of initial visits with new referrals to hospice, home health, or home caregiver agencies; percent of initial visits with the prescription of durable medical equipment.

### Analysis

We used descriptive statistics to report the demographics and quality standards for all patients, including the serious illness within the population and how well the REACH program functioned across the seven domains. Each standard was noted as completed only if it was noted to be completed in its entirety upon chart review, e.g., the entire VA-SLUMS was performed, all IADLS/ADLS were evaluated, a symptom was documented in the note as assessed. We noted the presence or absence of each standard within each domain, to capture the breadth of the domains. All statistical analyses were performed using STATA/SE 11.2 (StataCorp, College Station, TX) or SPSS for Windows Version 24 (IBM Corp., Armonk, NY).

## Results

### Characteristics of patients

Of the 159 patients enrolled in the REACH program during the study period, 56% were female, 63% were white and 33% black, 97% were English-speaking, and 42% were married. The mean age of the patients was 70 years (S.D. ± 17). Patients enrolled in the REACH program for 261 days on average (S.D. ± 181 days) with a median of 237 days (IQR 117–379) (Table 1). Fifty-three percent had 3 or more comorbidities per the Charlson Comorbidity Index. The most common comorbidities were dementia (32%), heart failure (31%) and moderate or severe renal disease (27%). Functionally, 45% had impairment in 2 or more ADLs. Overall, 31% of the population died in the follow-up period (Table 2). Over the study period, 8% discontinued the REACH program and 6% were admitted to a skilled nursing facility.

**Table 1:** Patient Characteristics, *n* = 159

Characteristics	N (%)
Age, mean (SD)	70.4 (17.0)
Female	89 (56)
Race	
White	100 (63)
Black	52 (33)
Other/not-reported	7 (4)
English-speaking	149 (97)
Marital Status	
Single, never married	10 (6)
Married	67 (42)
Divorced	24 (15)
Widowed	38 (24)
Other/ Unknown	20 (13)
Days enrolled in REACH program	261 (181)

**Table 2:** Serious Illness Characteristics, *n* = 159

Characteristics	N (%)
Charlson Comorbidity Score	
0	18 (11)
1–2	57 (36)
3–4	38 (24)
≥ 5	46 (29)
Number of chronic health conditions, mean (±SD)	3.2 (2.5)
Most Common Comorbidities	
Dementia	51 (32)
Congestive heart failure	50 (31)
Moderate or severe renal disease	43 (27)
Cognitive Impairment Score (VA-SLUMS), mean (±SD), *n*=55	18.1 (7.4)
ADL independence	
None	22 (14)
1–2 ADLs	26 (17)
3–4 ADLs	21 (14)
5–6 ADLs	85 (55)
Independent ADLs, mean (±SD)	3.9 (2.2)
Mortality during study period	50 (31)

### Standards of quality-of-care

Frequency of standards differed across the seven quality domains (Table 3). *Completion of medical assessment* on initial visits were 98% for functional assessment of IADLs/ADLs, 49% for communication barriers, 38% for cognitive impairment, and 22% for spiritual needs. *Care coordination* at the initial visit varied from 48% with communication to the patient’s primary care provider, 45% with communication among the REACH program team, to 25% with communication to a specialty clinician. For *safety* concerns, 98% had a falls assessment, 96% had a medication safety review, and 21% of patients had a behavioral issue assessment at their initial visit. The frequency of *quality-of-life* assessments, including symptom burden, ranged from 70% for pain, 57% for dyspnea and constipation, and 53% for psychosocial distress. *Clinician competency* measures revealed 59% of patients were assessed and brought up-to-date with influenza vaccination. Related to *clinician competency* in prescribing, 54% of patients with a diagnosis of depression were prescribed anti-depressants and 11% of patients were prescribed opiates (and of those, 68% received a bowel regimen. Related to *goal attainment,* surrogacy was discussed 73% of the time, and advance directives 65%. Related to *access*, REACH clinicians made home health referrals for 20% of patients, ordered durable medical equipment for 15% of patients, and placed hospice referrals during 7% of initial visits.

**Table 3:** Standards Assessed at Initial REACH Visit, *n* = 159

Domain	Standards Assessed	N (%)
Medical Assessments	IADLs/ADLs assessment	157 (97)
	Communication barriers assessment	79 (50)
	Formal cognitive assessment (VA-SLUMS)	55 (35)
	Spiritual needs assessment	35 (22)
Care Coordination	Communication with PCP	77 (48)
	Communication with specialist	39 (25)
	Communication with REACH social worker or pharmacist	71 (45)
Safety	Falls assessment	155 (98)
	Medication safety review	153 (96)
	Behavioral issue assessment	34 (21)
Quality-of-life	Pain assessment	112 (70)
	Dyspnea assessment	91 (57)
	Constipation assessment	91 (57)
	Psychosocial distress assessment	85 (53)
	Poor appetite assessment	61 (38)
	Depressive symptoms assessment	61 (38)
	Fatigue assessment	59 (37)
	Anxiety assessment	51 (32)
	Nausea assessment	42 (26)
Clinician competency	Influenza vaccination up-to-date	92 (59)
	Depression treatment, *n* = 61	33 (54)
	Opiate prescribed at initial visit	17 (11)
	Bowel regimen prescribed if indicated, *n* = 65^a^	44 (68)
	Antipsychotic prescribed at initial visit	6 (4)
	Benzodiazepine prescribed at initial visit^b^	4 (3)
Goal attainment	Surrogate discussed	116 (73)
	Advance directives discussed	103 (65)
Access	Home health referral placed at initial visit	32 (20)
	Durable medical equipment ordered	24 (15)
	Hospice referral placed at initial visit	5 (3)

^a^ *n*=65, 65 patients had a history of opiate use prior to the initial visit, of whom 13 received an opiate prescription at the initial visit. Four patients, who had not received an opiate prescription previously, received one at the initial visit. Of the 17 prescribed opiates at the initial visit, 12 received a bowel regimen and 1 had a contraindication for a bowel regimen

^b^ Of the 37 patients who had a history of benzodiazepine use prior to the initial visit, 3 received a prescription at the initial visit. One patient who had not received a benzodiazepine prescription previously, received one at the initial visit

## Discussion

The evaluation of the REACH program by quality-of-care domains demonstrated strengths and weaknesses in the program’s quality. After operationalizing proposed domains, the program had near universal evaluation of common serious-illness syndromes such as falls, but chart review found low rates of assessment for behavioral issues and others. These assessments are necessary first steps to improving symptom control, as demonstrated in other programs [26]. However our study is incomplete in that it fails to examine symptom control over time.

Similar to a recent study that found 50% of their patients had advance directives completed, advance care planning occurred in 65% of initial visits [11]. A randomized control trial of a home-based palliative care program found 71% of patients completed advance directives over the duration of the program [26]. This fact is reassuring given the impact of advance care planning on patient trajectories [27]. However, other important areas such as behavioral issues, cognitive barriers, or spiritual needs were less commonly assessed. A systematic review of home-based palliative care programs found no effects of these programs on behavioral issues, cognitive status, or spiritual well-being, perhaps because they are infrequent targets of such programs [28].

This study has several limitations including the limited generalizability of our results, which come from a single community-based serious-illness care program in central North Carolina, enrolled over a discrete period. We will need to validate our method for chart review. Significant challenges exist in implementing an EHR-based quality measurement such as time commitment and data fidelity [11]. Longitudinal work is needed to determine if the quality domains with low frequency of assessment were reviewed at later time points, and how best to assess these standards. A 1-month or 3-month window may be more appropriate when evaluating home-based quality metrics. The frequency of overall visits or urgent visits might also be an important metric and should be examined in future work. The presence or absence of other family or caregivers may also be important to the quality of these programs and should also be measured going forward. The ideal rates of assessment of some quality domains is unclear, and some of these assessments may not be indicated for all patients.

We could not develop chart-based standards for all of the domains as recommended by the National Home-Based Primary and Palliative Care Network [8]. As the field moves forward, quality-of-care will need more comprehensive measures of value that incorporate patient preference and patient-reported outcomes [13]. Those standards most recently proposed by Ritchie et al. are consistent with those used in this study and provide additional standards for all domains [24]. Goal attainment for patients is more expansive than simply completing an advance care planning form, and the field will need to move beyond recording the simple presence or absence of advance care planning in the chart. The quality of the advance care planning and patient satisfaction around the advance care planning are important and were unmeasured in this study. Further steps will include improving processes to pull data directly from the EHR, though chart review may still serve an important method to assess quality.

## Conclusions

This study is one of the first to operationalize and examine quality-of-care measures for a community-based serious-illness care program, an emerging model for vulnerable adults. We found the REACH program cared for patients similar to those in other community-based serious-illness care programs [5, 6, 29]. The patients were multi-morbid, functionally limited, and near the end-of-life. Our operationalization should not constitute validation of these measures and revealed areas for improvement; however, the community-based serious-illness care program performed well in several key quality-of-care domains. Future work is needed to validate these measures.

## Data Availability

The datasets used and/or analyzed during the current study are available from the corresponding author on reasonable request.

## Abbreviations

REACH program: Reaching out to Enhance the care of Adults in their Communities and their Homes
EHR: Electronic health record
CCI: The Charlson Comorbidity Index
IADLs: Instrumental Activities of Daily Living
ADLs: Activities of Daily Living
VA-SLUMS: Veterans Affairs St. Louis University Mental Status Exam
